# Case report: Recurrent pontine stroke and leukoencephalopathy in a patient with *de novo* mutation in *COL4A1*

**DOI:** 10.3389/fneur.2023.1237847

**Published:** 2023-09-27

**Authors:** Hui Zhang, Kai-Li Fan, Yue-Qi Zhang, Xiao-Yan Hao, Xiang-Zhen Yuan, Shu-Yun Zhang

**Affiliations:** ^1^Department of Neurology, Weifang People’s Hospital, Weifang, China; ^2^Department of Nephrology, Weifang People’s Hospital, Weifang, China; ^3^Weifang Center for Disease Control and Prevention, Weifang, China

**Keywords:** *COL4A1*, PADMAL, cerebral small vessel disease, stroke, case report

## Abstract

This report presents a case of pontine autosomal dominant microangiopathy with leukoencephalopathy (PADMAL) in a 35 year-old male patient. The patient exhibited a consistent history of recurrent ischemic strokes, concentrated primarily in the pons region, accompanied by concurrent manifestations of leukoencephalopathy and microbleeds. Genetic evaluation revealed a heterozygous missense mutation consistent with c.3431C>G, p. Thr1144Arg substitution within exon 40 of the *COL4A1* gene. This mutation was also identified in the patient’s mother, affirming an autosomal dominant inheritance model. Our findings serve as testament to the potential role of mutation in the exon 40 of *COL4A1* in the pathogenesis and progression of PADMAL, contributing to ongoing efforts aimed at better understanding the genetic basis of this debilitating disorder.

## Introduction

The alpha-1 chain of collagen type IV (COL4A1) constitutes a key constituent of the basement membrane of various vital organs throughout the human body. The extensive expression of this protein explains the highly heterogeneous and multifaceted spectrums of pathologies that arise from its genetic aberrations. Of particular note is the organ most susceptible to these genetic abnormalities—the brain. Specifically, phenotypes such as leukoencephalopathies, porencephaly malformations, cerebral small vessel diseases with hemorrhage, and hereditary angiopathy with nephropathy, aneurysm, and cramps (HANAC) have repeatedly emerged due to mutations in this gene (1). To further elucidate the complexities of *COL4A1*-related diseases, Ding et al. reported the novel presentation of pontine infarction and leukoencephalopathy in a pedigree, disengaging itself from the more common cerebral autosomal dominant arteriopathy with subcortical infarcts and leukoencephalopathy (CADASIL). These unique clinical features prompted the definition of a new autosomal dominant disease, termed pontine autosomal dominant microangiopathy with leukoencephalopathy (PADMAL) (2). Subsequent investigations established that mutations within the 3′ untranslated region (UTR) of *COL4A1* were responsible for the onset of PADMAL. These genetic events impede the binding of microRNA-29 and, as a consequence, activate the expression of *COL4A1* (3). In the present case report, we present a young male patient with PADMAL, who has a missense mutation localized to exon 40 within *COL4A1*. These findings contribute to a greater understanding of the clinical complexities of PADMAL and further highlight the interplay between gene regulation, genetic aberration, and neurological disease etiology.

## Case report

A 35 years-old male patient comes to our department with the sudden onset of dysarthria, dysphagia, and mild hemiplegia of the right limb. The patient developed dysarthria 2 years ago and was diagnosed with pontine infarct in a local hospital. One year ago, the patient came to our hospital with dysarthria and limb weakness. Magnetic resonance imaging (MRI) showed acute infarcts in bilateral frontal lobes and corona radiata, multifocal lacunae in the cerebral hemispheres and brain stem, and leukoencephalopathy. The patient was diagnosed with cerebral small vessel disease (cSVD) and the pathogenesis was unclear.

**Figure 1:**
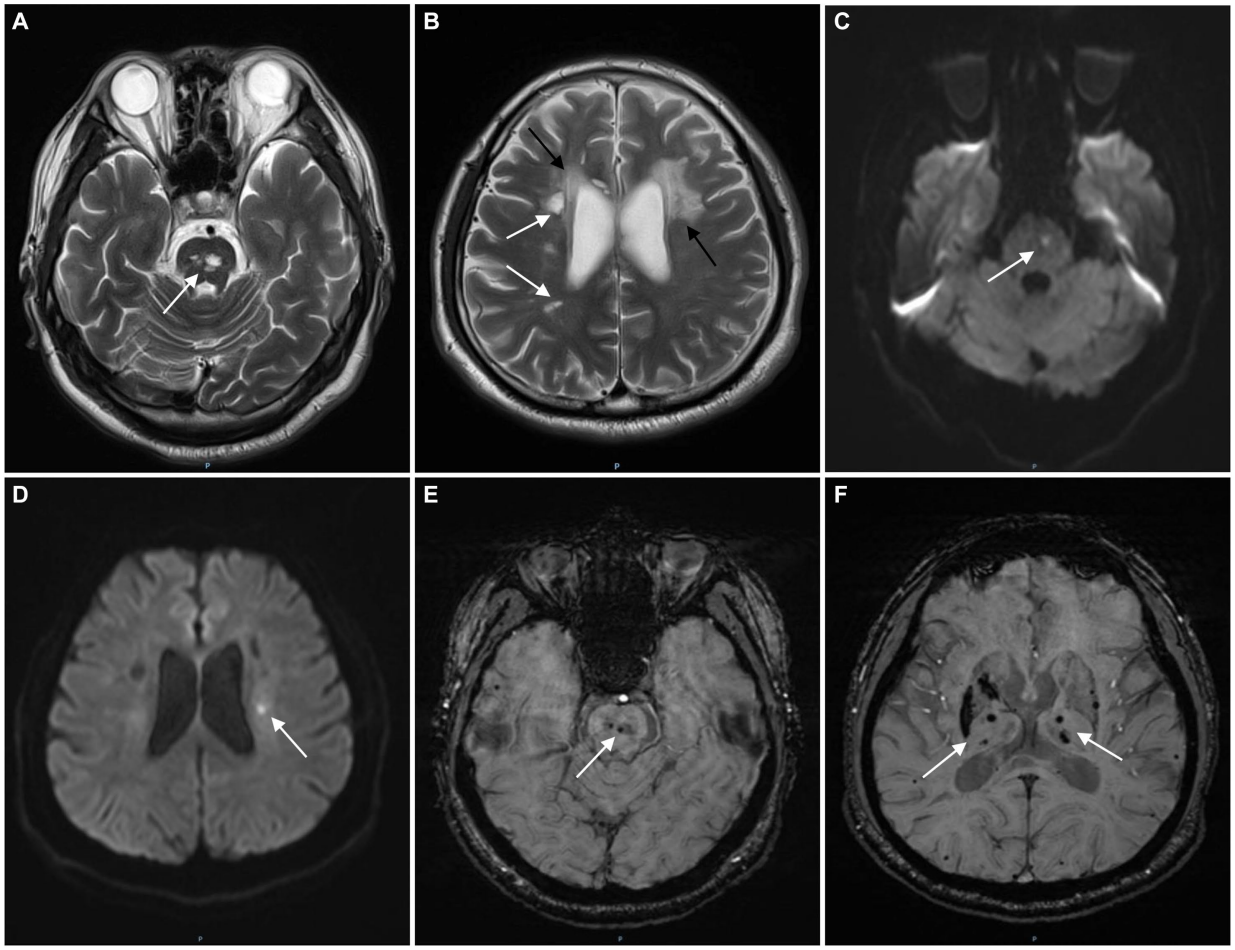
The magnetic resonance imaging (MRI) images of the patient. T2-weighted image shows multifocal lacunes in the pons (**A**, white arrow) and periventricular white matter (**B**, white arrows) and leukoencephalopathy (**B**, black arrows). Diffusion weighted imaging (DWI) shows acute infarcts in the pons (**C**, white arrow) and left corona radiata (**D**, white arrow). Multifocal microbleeds and hemosiderin deposits were found in the pons (**E**, white arrow), bilateral thalamus and basal ganglia (**F**, white arrows) on susceptibility weighted imaging (SWI).

**Figure 2:**
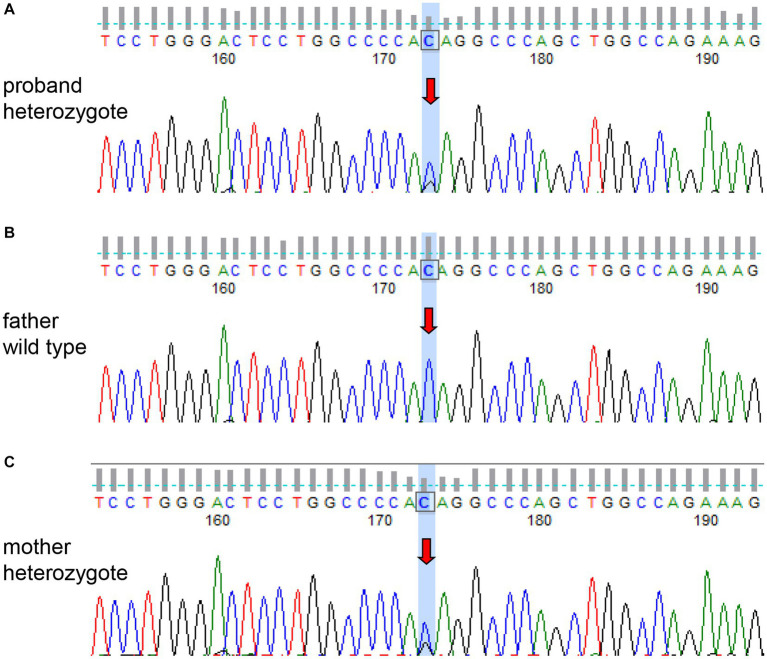
Sanger sequencing of the *COL4A1* gene of the patient and his parents. The patient **(A)** and his mother **(C)** had the same heterozygous mutation (c.3431C>G). The genotype of the patient’s father was wild type **(B)**.

The patient had a full-term vaginal birth with normal development and finished his middle school. According to the patient’s statement, his maternal grandfather died of illness in his 30s and his maternal grandmother died of cerebral infarction in her 80s, but no further information could be provided. None of the five siblings of the patient’s mother had a history of cerebrovascular disease. Mild cognitive impairment was observed, and the scores of Montreal Cognitive Assessment (MOCA) and mini-mental state examination (MMSE) were 21 and 25, respectively. No significant risk factors for cerebrovascular disease were found. Laboratory tests showed that blood lipids, blood glucose, and blood homocysteine were within normal limits. We further tested thyroid function, rheumatoid factor, erythrocyte sedimentation rate, complement C3 and C4, immunoglobulin, anticardiolipin antibody, antineutrophil cytoplasmic antibody, antinuclear antibody spectrum, and found no obvious abnormalities. Renal ultrasound and cardiac ultrasound were normal. Ophthalmic examination revealed normal retinal vasculature. Brain MRI showed acute cerebral infarcts in the pons and left corona radiata, multiple lacunae in the pons, subcortical white matter (WM) and periventricular WM, multifocal microbleeds in the pons, bilateral thalamus and basal ganglia, and leukoencephalopathy (Figure 1). Magnetic resonance angiography (MRA) found no significant abnormalities in the cerebral arteries. Whole exome sequencing was performed using Illumina HiSeq platform and a heterozygous missense mutation in exon 40 of *COL4A1* (chr13:110826321, c.3431C>G, p. Thr1144Arg) was found. No mutations were found in *ABCC6*, *APP*, *COL3A1*, *COL4A2*, *COLGALT1*, *CST3*, *FOXC1*, *GLA*, *HTRA1*, *NOTCH3* and *TREX1*, which were reported to be associated with cSVD. To further confirm the genotype of the patient’s parent, *COL4A1* gene was tested using Sanger sequencing. The heterozygous mutation c.3431C>G in the exon 40 of *COL4A1* was identified in the patient’s mother (Figure 2). To our knowledge, the variant has not been reported in patients with cSVD and has been interpreted as uncertain or benign in the ClinVar database.^1^ Although the patient’s mother carried this variant, she had no history of cerebrovascular disease, and she declined further MRI scans of the brain.

The function of the missense mutation (p. Thr1144Arg) was evaluated using REVEL, ClinPred, SIFT and Polyphen-2 software. The REVEL software score was 0.237 (greater than 0.75 is predicted to be harmful). ClinPred software score was 0.1412 (greater than 0.5 is predicted harmful). SIFT and Polyphen-2 software were used to predict the protein function, and the results were harmless and harmful, respectively. Conservation analysis showed that the amino acids at this site were highly conserved across species, suggesting the mutation p. Thr1144Arg may be potentially pathogenic (details can be found in Supplementary material).

The patient was diagnosed with PADMAL, according to previous literature reports (2). After treatment with edaravone (60 mg per day), citicoline sodium (300 mg per day), acupuncture, physical and speech rehabilitation training for 2 weeks, the symptoms of dysarthria and right hemiplegia were significantly improved. One month later, the patient’s symptoms were basically relieved, and the modified Rankin scale (mRS) score was 1 point.

## Discussion and conclusion

*COL4A1* gene is located on the 13q34 chromosome and contains 52 exons, which encodes the α1 chain of collagen IV. Collagen IV is a key component of the basement membrane and its structural changes can affect the stability of the vascular basement membrane, resulting in ischemic or hemorrhagic diseases. In *COL4A1*-related diseases, most of the mutations were missense mutations, which tend to affect the glycine-X-Y repeats in the tri-spiral domain of α1 chain and the folding and secretion of collagen IV. However, missense mutations involving non-glycine residues of the triple-helix were also reported (4). The substitution of highly conserved residues in the triple-helical domain is assumed to change the whole heterotrimer structure, which may affect the secretion of heterotrimers in the matrix and finally lead to structural or functional abnormalities of basement membranes (5). In this case, the young male patient had recurrent ischemic stroke and leukoencephalopathy, and the pons were significantly affected, which was quite different from CADASIL. We did not find risk factors and other mutations in cSVD-associated genes in the patient. Therefore, we hypothesized that the missense mutation in exon 40 of *COL4A1* (c.3431C>G) was responsible for recurrent stroke and pathological changes in the brain. The patient mainly presented with repeated infarcts in the pons, consistent with the manifestations of PADMAL, and there were also multifocal lacunae in the basal ganglia and WM. In addition to dysarthria, mild cognitive impairment, particularly executive dysfunction, was found in this patient. However, the results of different bioinformatics analysis software for this mutation were not consistent. There is still no strong functional or bioinformatic evidence that the mutation can cause PADMAL. Therefore, the pathogenicity of the mutation needs to be validated in a larger population or through more in-depth bioinformatics analysis.

Most *COL4A1* mutations are autosomal dominant inheritance, but the phenotypic spectrum is highly heterogeneous. Moreover, penetration of *COL4A1* mutations is rather incomplete, suggesting that modifying factors may be involved (5). In this case report, the mutation in the *COL4A1* of the patient was inherited from his mother, who was asymptomatic and had no history of cerebrovascular disease. The incomplete penetrance of *COL4A1* has been reported in several other pedigrees (6, 7). However, the mechanisms of incomplete penetrance and modifying factors are still unclear. Currently, there is no effective treatment for *COL4A1*-related diseases. These patients with ischemic stroke also have a tendency to hemorrhage. As seen in the patient, we reported, there were large numbers of cerebral microbleeds. Antithrombotic or anticoagulant therapy may increase the risk of bleeding and is not recommended in *COL4A1*-related cSVD (8).

In summary, for young patients with recurrent pontine infarcts and leukoencephalopathy, mutations in *COL4A1* should be considered. Not only mutations in the 3′ UTR but also in the exons of *COL4A1* may cause PADMAL. Although PADMAL is an autosomal dominant disorder caused by *COL4A1* mutations, the penetration of *COL4A1* mutations is rather incomplete.

## Data availability statement

The original contributions presented in the study are included in the article/Supplementary material, further inquiries can be directed to the corresponding author.

## Ethics statement

The requirement of ethical approval was waived by Medical Ethics Committee of Weifang People’s Hospital. The studies were conducted in accordance with the local legislation and institutional requirements. The participants provided their written informed consent to participate in this study. Written informed consent was obtained from the individual(s) for the publication of any potentially identifiable images or data included in this article.

## Author contributions

X-ZY and S-YZ: study design. HZ, K-LF, X-YH, and Y-QZ: data collection and analysis. HZ: original draft writing. X-ZY and Y-QZ: review and editing. All authors contributed to the article and approved the submitted version.

## Footnotes

^1^ https://www.ncbi.nlm.nih.gov/clinvar
