# Supplementary material for: Case report: Recurrent pontine stroke and leukoencephalopathy in a patient with de novo mutation in COL4A1
Source: Front Neurol. 2023 Sep 27;14:1237847. doi: 10.3389/fneur.2023.1237847 (PMC10564987; doi:10.3389/fneur.2023.1237847)
Supplement: Supplementary file 1 [file Table_1.DOCX]

## Supplementary Materials


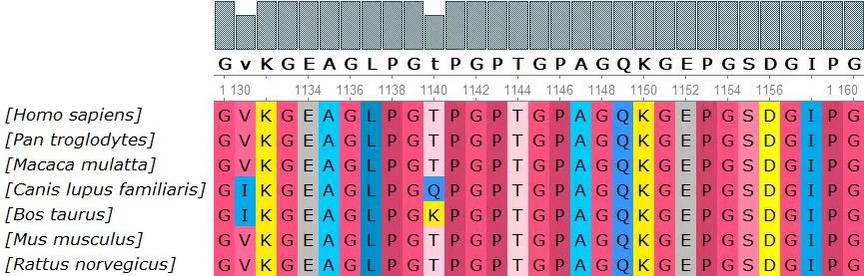


**Figure S1** Conservation analysis showed that the amino acid at the site of 1144 was highly conserved across species. Sequence information was derived from the NCBI-Protein database.
